# Clinical characteristics, treatment, and survival of 30 patients with gastrointestinal natural killer/T‐cell lymphoma

**DOI:** 10.1002/cnr2.1800

**Published:** 2023-03-15

**Authors:** Jia‐Xin Liu, Xin Liu, Yong Yang, Wei‐Ping Liu, Ying Wang, Xia He, Li‐Ling Zhang, Bao‐Lin Qu, Li‐Ting Qian, Xiao‐Rong Hou, Xue‐Ying Qiao, Hua Wang, Gao‐Feng Li, Yuan Zhu, Jian‐Zhong Cao, Jun‐Xin Wu, Tao Wu, Su‐Yu Zhu, Mei Shi, Hui‐Lai Zhang, Hang Su, Yu‐Jing Zhang, Jun Zhu, Shu‐Nan Qi, Ye‐Xiong Li, Yu‐Qin Song

**Affiliations:** ^1^ Key Laboratory of Carcinogenesis and Translational Research (Ministry of Education), Department of Lymphoma Peking University Cancer Hospital & Institute Beijing China; ^2^ National Cancer Center/Cancer Hospital, Chinese Academy of Medical Sciences (CAMS) and Peking Union Medical College (PUMC); Center for Cancer Precision Medicine, CAMS and PUMC National Institute of Biological Sciences, Collaborative Innovation Center for Cancer Medicine Beijing People's Republic of China; ^3^ Fujian Medical University Union Hospital Fuzhou China; ^4^ Chongqing University Cancer Hospital & Chongqing Cancer Hospital Chongqing China; ^5^ Jiangsu Cancer Hospital & Jiangsu Institute of Cancer Research Nanjing Jiangsu China; ^6^ Union Hospital, Tongji Medical College Huazhong University of Science and Technology Wuhan Hubei China; ^7^ The General Hospital of Chinese People's Liberation Army Beijing China; ^8^ The Affiliated Provincial Hospital of Anhui Medical University Hefei Anhui China; ^9^ Peking Union Medical College Hospital, Chinese Academy of Medical Sciences (CAMS) and Peking Union Medical College (PUMC) Beijing China; ^10^ The Fourth Hospital of Hebei Medical University Shijiazhuang China; ^11^ Second Affiliated Hospital of Nanchang University Nanchang China; ^12^ National Geriatric Medical Center Beijing Hospital Beijing China; ^13^ Cancer Hospital of the University of Chinese Academy of Sciences Zhejiang Cancer Hospital Zhejiang Hangzhou China; ^14^ Shanxi Cancer Hospital and the Affiliated Cancer Hospital of Shanxi Medical University Taiyuan Shanxi China; ^15^ Fujian Provincial Cancer Hospital Fuzhou Fujian China; ^16^ Affiliated Hospital of Guizhou Medical University Guizhou Cancer Hospital Guiyang Guizhou China; ^17^ Hunan Cancer Hospital and the Affiliated Cancer Hospital of Xiangya School of Medicine Changsha Hunan China; ^18^ Xijing Hospital of Fourth Military Medical University Xi'an China; ^19^ Key Laboratory of Cancer Prevention and Therapy, National Clinical Research Center for Cancer Tianjin Medical University Cancer Institute & Hospital Tianjin China; ^20^ The Fifth Medical Center of PLA General Hospital Beijing China; ^21^ Sun Yat‐Sen University Cancer Center State Key Laboratory of Oncology in South China; Collaborative Innovation Center for Cancer Medicine Guangzhou Guangdong China

**Keywords:** asparaginase/therapeutic use, efficacy, physiopathology, retrospective analysis, T cell lymphoma

## Abstract

**Background:**

The gastrointestinal (GI) tract is the second most frequent extranasal involvement site for ENKTL. This study aimed to explore the clinicopathological features, treatment models, survival outcomes, and prognosis of gastrointestinal ENKTL (GI‐ENKTL).

**Methods:**

The clinical data of GI‐ENKTL patients were extracted from the China Lymphoma Collaborative Group (CLCG) database and were analyzed retrospectively.

**Results:**

A total of 30 patients were enrolled, with a male/female ratio of 4:1 and a median age of 42 years. Twenty‐nine patients received chemotherapy, of whom 15 patients received asparaginase‐based (ASP‐based) regimens. Moreover, seven received surgery and three received radiotherapy. The overall response an d complete remission rates were 50.0% and 30.0% for the whole cohort, 50.0% and 37.5% for patients treated with ASP‐based regimens, and 50.0% and 25.0% for those treated with non‐ASP‐based regimens, respectively. The median follow‐up was 12.9 months and the 1‐year overall survival rate was 40.0% for the whole cohort. For those patients in an early stage, ASP‐based regimens resulted in a superior 1‐year progression‐free survival rate compared to non‐ASP‐based regimens (100.0% vs. 36.0%, *p* = .07). However, ASP‐based regimens did not improve survival in patients at an advanced stage.

**Conclusion:**

GI‐ENKTL still has a poor prognosis, even in the era of modern asparaginase‐based treatment strategies.

## 1 INTRODUCTION

Extranodal natural killer (NK)/T‐cell lymphoma (ENKTL) comprises a subgroup of cytotoxic T or NK cells that originate outside the lymph nodes and is associated with Epstein–Barr virus.^1^ ENKTL has a strong geographic presence in Latin American and East Asian populations (accounting for 5.0%–15.0% of all lymphomas) and is rare in European countries and America (less than 1% of all lymphoma).^2^, ^3^, ^4^, ^5^, ^6^, ^7^, ^8^ ENKTL is almost exclusively extranodal, with 80.0%–90.0% of cases involving the upper aerodigestive tract (UADT), including the nasal cavity, paranasal sinuses, nasopharynx, Waldeyer's ring, and palate. Cases of extranasal areas commonly occur in the skin/soft tissues and the gastrointestinal (GI) tract, followed by the testis, lungs, adrenal glands, and the central nervous system.^4^

The outcome of ENKTL patients is affected by multiple factors and tumor location is a determining factor in tumor malignancy. Although the GI tract is the most common anatomic site of extranodal non‐Hodgkin's lymphoma that accounts for 6.0%–23.0% of all NHLs and 0.9%–6.5% of all GI cancers,^7^ information on ENKTL with GI tract (GI‐ENKTL) involvement is relatively scarce. According to a previous study based on 81 patients from the Asia Lymphoma Study Group in 2013, GI‐ENKTL presented with more severe clinical features and had an extremely poor prognosis.^8^ However, the study did not evaluate the widely used ASP‐based regimens that have been developed in recent years. In addition, previous studies had small sample sizes, were from a single institution, and had incomplete clinic data.^9^, ^10^, ^11^, ^12^

We, therefore, retrospectively analyzed the clinical features, treatment strategies, and outcomes of patients with GI‐ENKTL using the database from the China Lymphoma Collaborative Group (CLCG). The aim was to evaluate the impact of different treatment modalities on outcomes in GI‐ENKTL.

## 2 METHODS

### 2.1 Patients

A total of 2640 patients with newly diagnosed ENKTL treated at 16 Chinese institutions between 2000 and 2016 in the CLCG database were retrospectively reviewed. The eligibility criteria included patients whose examination revealed GI tract involvement. Patients who were lost to follow up or had incomplete data were excluded. Lymphomatoid gastropathy (LyGa) pathologically confirmed has been excluded by hematopathologists. Patients of all ages and both sexes were considered for inclusion. Eventually, 30 patients formed the study population. Follow‐ups were conducted via an outpatient clinic and over telephone. Routine follow‐up plan includes physical examinations and imaging every 3 months during the first 3 years, twice a year during the fourth and fifth year, and annually after 5 years. No patients were lost to follow‐up.

The staging work‐up of ENKTL included a routine physical examination, chest/abdominal/pelvic computed tomography (CT) or positron emission tomography (PET)‐CT scan, nasopharyngeal biopsies, and bone marrow aspirate analyses. For cases undergoing surgery, the removed specimen tissues were used for staging. Patients who filled the following criteria were included: (1) patients who presented initially with GI symptoms with ruled out aggression in other distant parts; and (2) patients with UADT lesions who were assayed for GI tract problems at the staging work‐up.

The SEER database was searched including the terms Histology recode; NK/T‐cell lymphoma, primary site, and nasal type; and the digestive system (label: C16.0‐C26.9). Individual details of 35 cases registered from 2000 to 2016 were retrieved using the SEER*Stat software (Version 8.3.6—August 8, 2019; Cancer Statistics Branch, NCI, Bethesda, MD). The following variables were analyzed: sex, year of diagnosis, age at diagnosis, Ann Arbor stage, treatment strategies, cause of death, and overall survival (OS).

### 2.2 Evaluation and definition

Clinical data, including demographics, stage, tumor location, serum lactate dehydrogenase (LDH), B symptoms, and Eastern Cooperative Oncology Group performance status (ECOG PS) were analyzed. With end‐of‐treatment evaluation after first‐line therapy including endoscopy with visual inspection, imaging analysis (CT or PET‐CT) according to the type of examination at the initial work‐up, and treatment modalities, including surgery, chemotherapy regimens, and radiotherapy, were also analyzed. The Ann Arbor staging and treatment evaluation were performed by local investigators following institutional imaging protocols in accordance with local standard practice.

Baseline disease characteristics, treatment strategies and patient outcomes for patients in the entire cohort were obtained from medical records. Responses were assessed using Lugano or Cheson criteria by PET or CT scans, clinician assessment, or both.^13^ Progression‐free survival (PFS) was defined as the time from first treatment to disease progression, relapse, or death. The OS was measured from the date of first treatment to the date of last follow‐up or death.

Prognostic stratification models were analyzed including the International Prognostic Index (IPI; including LDH level, stage, B symptoms, and regional lymph node involvement) as well as the nomogram‐revised risk index (NRI), a model derived from the international multicenter analysis of data from 1582 patients receiving asparaginase treatment. One point was assigned to each age >60 year, LDH, stage II disease, ECOG score ≥2, or primary tumor invasion. Two points were assigned to stage III/IV. Patients were then stratified into four risk groups (low, 0; intermediate low, 1; intermediate high, 2; and high, ≥3).^14^

### 2.3 Follow‐up and statistical analysis

Follow‐ups were conducted via an outpatient clinic and over telephone. Routine follow‐up plan includes physical examinations and imaging every 3 months during the first 3 years, twice a year during the fourth and fifth year, and annually after 5 years. No patients were lost to follow‐up

Over survival (OS) and PFS were estimated using the Kaplan–Meier curves and log‐rank test was to assess the long‐term prognosis stratified by chemotherapy regimens, prognostic factors and prediction models. Variables in the univariate analysis and other factors that had potential clinical significance were further entered into the Cox regression analysis to analyze independent risk factors. To compare the characteristics of patients, Fisher's exact test (or *χ* ^2^ test) was used. A two‐sided *p*‐value of <.05 was considered to indicate statistical significance. All statistical analyses were performed using SPSS (IBM SPSS Statistics for Macintosh, Version 25.0).

## 3 RESULTS

### 3.1 Patients' characteristics

The clinical features of all patients are shown in Table 1. Their median age was 42 years (16–84 years). There was a male predominance with a male/female ratio of 4:1. The proportion of advanced disease was over 50.0%, however, five (16.7%) patients had poor performance status (ECOG PS ≥2), of which the possible cause was the patient were relatively young. In the IPI model, more than half of the patients (*n* = 16, 53.3%) were in the low‐risk IPI category for ECOG PS <2 and age <60 years.

**TABLE 1:** Characteristic features of patients with GI‐ENKTL

Characteristics	*N* (%)	*p*
Age (y)		
≤60	27 (90)	>.05
>60	3 (10)	
Sex		
Male	24 (80)	>.05
Female	6 (20)	
B symptoms		
Absence	13 (43)	>.05
Presence	17 (57)	
ECOG PS		
<2	25 (83)	>.05
≥2	5 (17)	
Extranodal sites of disease		
<2	19 (63)	.007
≥2	11 (37)	
Ann Anbor stage		
I/II	11 (37)/3 (10)	.002
III/IV	16 (50)	
Serum LDH		
Normal	20 (67)	.021
Increased	10 (33)	
IPI		
0–1	16 (53)	.001
2	9 (30)	
≥3	5 (17)	
NRI		
0–1	10	.001
2	3	
≥3	17	

Abbreviations: ECOG PS, Eastern Cooperative Oncology Group performance status; IPI, the International Prognostic Index; LDH, lactate dehydrogenase; NRI, the nomogram‐revised risk index.

**FIGURE 1:**
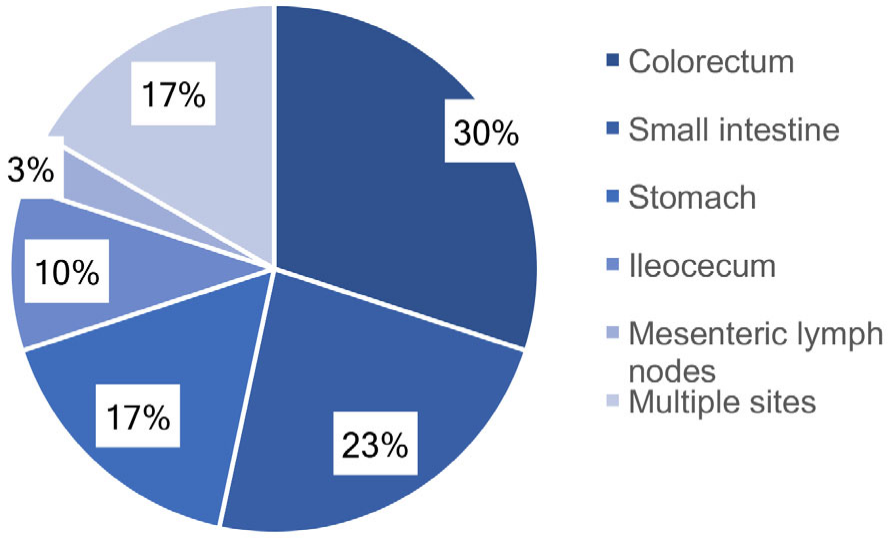
Distribution of the anatomic involvement of the digestive tracts. Proportion of each part in 30 patients with gastrointestinal (GI) involvement

The specimens for pathologic diagnosis were obtained from biopsies collected with colonoscopy (*n* = 23; 76.7%) or surgically removed primary masses (*n* = 7; 23.3%). Based on the GI tract involved sites, the most common site was the colon (*n* = 9; 30.00%), followed by the jejunoileum (*n* = 7; 23.33%), stomach (*n* = 5; 16.67%), ileocecum (*n* = 3; 10.00%), and mesenteric lymph nodes (*n* = 1; 3.33%). Five (16.67%) patients presented with multiple involved sites (Figure 1).

### 3.2 Treatment models

Radiotherapy could be radical treatment for limited‐stage ENKTL originating from UADT. However in this GI‐ENKTL cohort, radiotherapy (*n* = 3; 10.0%) was usually performed for salvage purposes and applied less frequently than chemotherapy and surgery, with a median radiotherapy dose of 45 Gy (27–52 Gy).

The main treatment modality for gastrointestinal lymphoma patients was chemotherapy. Among 29 patients who were treated with systematic chemotherapy, 16 patients were treated with ASP‐based regimens, such as CHOPL (cyclophosphamide, vincristine, doxorubicin, and prednisone, L‐asparaginase), P‐GEMOX (gemcitabine, oxaliplatin, and pegaspargase), LOP (L‐asparaginase, vincristine, and prednisone), and SMILE (dexamethasone, methotrexate, ifosfamide, L‐asparaginase, and etoposide), whereas 13 patients were treated with non‐ASP‐based regimens. Most of these treatments were based on anthracyclines, such as CHOPE (cyclophosphamide, vincristine, doxorubicin, prednisone, and etoposide), CHOP (cyclophosphamide, vincristine, doxorubicin, and prednisone), and EPOCH (etoposide, prednisone, vincristine, cyclophosphamide, and doxorubicin). The median chemotherapy cycles of ASP‐based and non‐ASP‐based regimens were 4 and 5, respectively.

**FIGURE 2:**
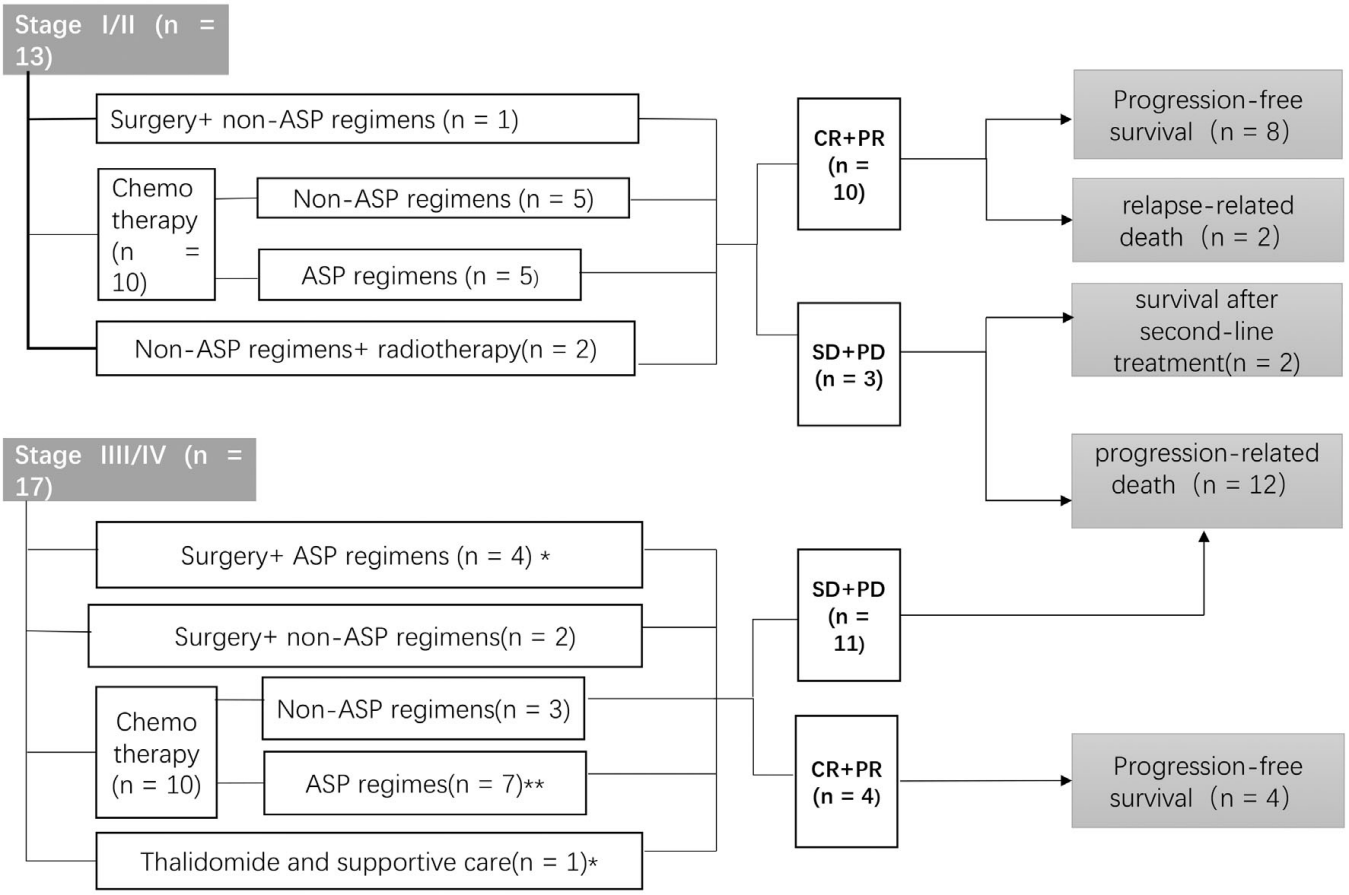
Summary of treatment approaches and outcomes. ASP regimens, asparaginase/pegaspargase‐containing regimens. *Evaluation of one case is unknown. **One patient received radiotherapy

Besides surgery was also an important strategy. Seven patients underwent surgeries for clinical manifestations, such as intestinal obstruction, bleeding, and perforation. Thus, the patterns of initial treatment were summarized in Figure 2.

In the cohort, high‐dose therapy and autologous stem cell transplantation (HDT‐ASCT) was given to one patient at stage I as consolidation therapy after CR in first‐line treatment.

### 3.3 Outcomes

Among 28 patients with available responses, nine patients (32.1%) reached CR and 5 (17.8%) achieved PR, while four patients (14.3%) remained at SD, and 10 (35.7%) had PD. Six patients in the ASP‐based regimens group (*n* = 15) reached CR and three patients reached PR, the CR and ORR rates were 40.0% and 60.0%, respectively. For the non‐ASP‐based regimens group (*n* = 13), the CR and ORR rates were 23.1% (3/13) and 46.1% (6/13), respectively. And no significant differences of CRR or ORR between the two groups were observed.

**FIGURE 3:**
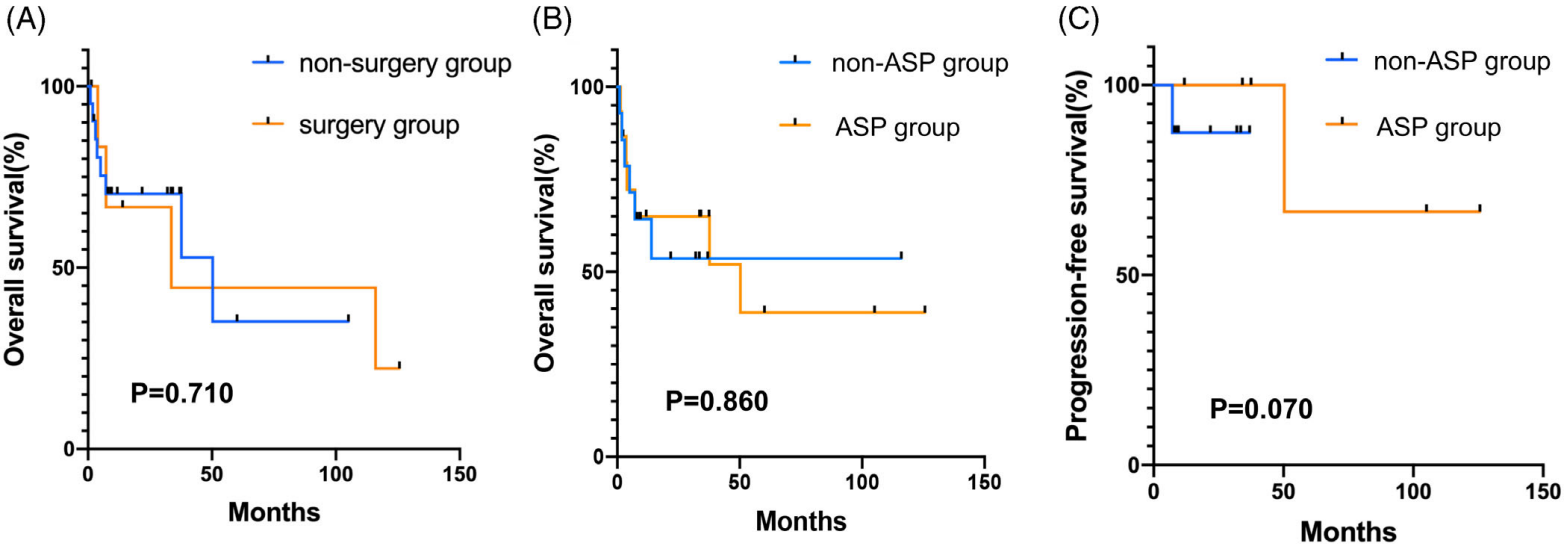
K‐M survival curves of long‐time outcomes according to treatments. (A) The comparison of overall survival showed no differences between the surgery group and non‐surgery group. (B) Comparison of overall survival based on the type of chemotherapy regimen. The old chemotherapy region versus new chemotherapy region showed no difference. (C) Comparison of progression‐free survival for patients in early stages based on the type of chemotherapy regimen, the asparaginase/pegaspargase (ASP) group showed a better trend than the non‐ASP group

The median follow‐up was 12.9 months (0.9–125.7 months). Fifteen patients (50.0%) had disease progression and 13 patients (43.3%) were dead at the last follow‐up. The median OS and the median PFS were 50.3 and 11.8 months, respectively. The 1‐ and 3‐year OS were 64.3% and 26.3%, respectively, and the 1‐ and 3‐year PFS were 48.0% and 17.2%, respectively. Surgery plus chemotherapy did not significantly improve survival in comparison with non‐surgery (*p* = .710, Figure 3A). In terms of chemotherapy regimens, the difference in OS rates between ASP‐based and non‐ASP‐based regimens did not show a statistically significant difference, with 1‐year OS and 3‐year OS rates of 66.7% versus 66.7% for the ASP‐based regimens, and 64.3% versus 57.1% for the non‐ASP‐based regimens (*p* = .860, Figure 3B). For early‐stage patients, ASP regimens (*n* = 5) resulted in a superior 1‐year PFS compared to non‐ASP‐based regimens (*n* = 8), with 1‐year PFS rate of 100.0% versus 36.0% (*p* = .070, Figure 3C). However, the OS was not significantly different between the two groups, with 1‐year OS rate of 100.0% for the ASP regimens and 87.5% for the non‐ASP‐based regimens (*p* = .390).

### 3.4 Prognostic factors and prediction models

**FIGURE 4:**
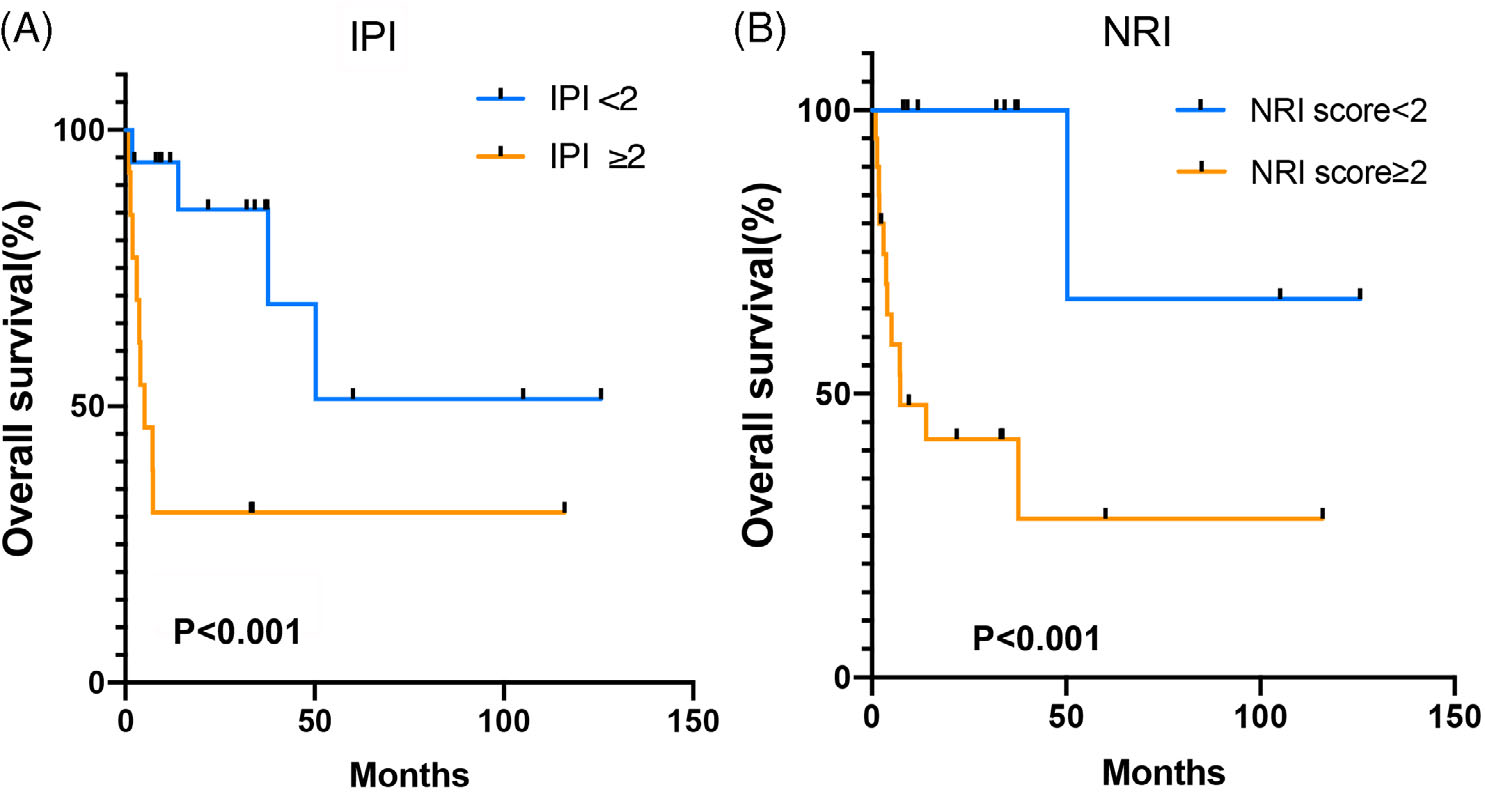
K‐M survival curves according to prognostic models. (A). Patients with an International Prognostic Index (IPI) score ≥2 had significantly lower survival (*p* < .001). (B). Patients with a nomogram‐revised risk index (NRI) score ≥2 had significantly lower survival (*p* < .001)

Univariate analysis revealed that an elevated LDH (*p* = .037), stage III‐IV (*p* = .003), and ≥2 extranodal involvement sites (*p* = .013) were statistically associated with poor OS, which was not associated with age >60 years (*p* = .713), and B symptoms (*p* = .683, Table 2). The COX analysis indicated that stage III/IV was an independent factor associated with poor survival (*p* = .009, HR = 2.004, 95% CI = 1.633–33.695, Table 2). The prognostic models, IPI score, and NRI score ≥2 showed a significant association with poor OS (Figure 4).

**TABLE 2:** Prognostic factors of GI‐ENKTL

	Univariate analysis			Multivariable analysis		
	*N*	Survival rate (%)	*p* Value	HR	95% CI	*p* Value
Stage I‐II	14	85.7	.003^†^	1		
Stage III‐IV	16	68.6		2.004	1.633–33.695	.009^†^
Normal LDH	20	70.0	.037^†^			
Elevated LDH	10	33.3				
<2 extranodal sites	19	78.9	.013^†^			
≥2 extranodal sites	11	37.5				
>60 years	3	66.7	.713			
≤ 60 years	27	55.5				
B symptoms	17	52.9	.683			
No B symptoms	13	61.5				

Abbreviations: 95% CI, 95% confidence interval; HR, hazard ratio; LDH, lactate dehydrogenase.

^†^ *p* < .05.

### 3.5 Comparison of GI‐ENKTLs and SEER database

A total of 35 GI‐ENKTL patients were identified in the SEER database. Among the 23 mortalities, 22 individuals succumbed to disease recurrence and one individual succumbed to comorbidity. The 1‐year OS rate was 30.3%

## 4 DISCUSSION

This was a retrospective Chinese multicenter study that aimed to analyze the characteristics and treatment of GI‐ENKTL. Accordingly, data from the T Project showed that the OS rate of ENKTL with extranasal disease at 3 years was 44% and at 5 years was 34%.^15^ In our cohort, the progress of GI‐ENKTL was worse, and the SEER database over the same period also demonstrated the same trend. There were three possible reasons for this: (1) most patients were in the advanced stage; (2) the GI lesions were prone to bleeding and perforation; and (3) the application of radiotherapy was unusual with only three people (10.0%) having received radiotherapy. Inferior prognostic indicators may include elevated LDH, late‐stage, and ≥2 extranodal involvement sites, with late‐stage being a strong indicator for failure and death, which is consistent with other studies.^12^ IPI and NRI prediction models were also applicable to GI‐NKTL patients.

Because it is a rare disease, there are few studies discussing the clinical features and survival outcomes according to chemotherapy regimens of GI‐ENKTL.^8^, ^9^, ^11^, ^12^ Chemotherapy is the most basic treatment modality. Accordingly, anthracycline‐based regimens were conventionally used in the past, but the efficacy was unsatisfactory, probably due to multidrug resistance caused by the expression of P‐glycoprotein.^16^ Under these circumstances, new non‐anthracycline agents that were not affected by P‐glycoprotein may improve response rates. Currently, ASP‐based (L‐asparaginase/pegaspargase‐based) regimens are recommended as first‐line chemotherapy for ENKTL.^17^, ^18^, ^19^ L‐asparaginase (L‐ASP) produces an anticancer effect by hydrolyzing and exhausting serum asparagine in NK/T‐cell lymphoma cells. Thereby such cells are unable to synthesize adequate proteins, RNA, and DNA, resulting in apoptosis. Pegaspargase, a glycol‐modified version of L‐ASP, with the advantages of lower immunogenicity and a longer half‐life, made it more widely available in regimens such as P‐GEMOX. Although both demonstrated safety and effectiveness in NKTLs,^20^, ^21^ there are few relevant reports about efficacy in GI‐involved cases.

In this research, despite ASP‐based regimens not showing better progress than non‐ASP‐based regimens in all cases, ASP‐based regimens in stage I/II showed an encouraging trend by achieving longer PFS, with the 1‐year PFS being 100.0% versus 36.0% (*p* = .070), which indicated superior outcomes in early‐stage patients. Even using a small sample size (*n* = 14), the validity of ASP‐based regimens was observed, showing the effect was meaningful. Larger sample sizes need to be studied for validation. In late‐stage patients, the proportion of patients receiving ASP‐based regimens was higher (11/17; 64.7%), but only two patients achieved CR, and four patients developed PD. Moreover, survival curves failed to show benefits compared to non‐ASP‐based regimens, with the 1 year PFS rate at 25.0% versus 42.9% (*p* = .608). Thus, the ASP‐based regimens were less effective in advanced patients with GI involvement. It is therefore necessary to find more effective drugs employing other mechanisms.

The role of surgery in therapeutic modalities for gastrointestinal lymphoma has been a matter of debate.^5^, ^22^, ^23^, ^24^, ^25^, ^26^ In a discrepancy that might be caused by a staging bias in that six of the seven patients undergoing surgery were in the advanced stage, a multicenter retrospective study on GI‐ENKTL showed significant beneficial effect of surgery,^5^ whereas surgery did not demonstrate a survival benefit in this study. Since these patients were prone to hemorrhage, perforation, and other complications, surgery was an essential approach to treat and prevent emergency complications such as bleeding and perforation. The group of patients undergoing elective surgery before complications had better outcomes compared to those undergoing emergency surgery due to complications in previous studies.^12^ Thus, choosing the right timing for surgery was also conducive to better outcomes. Therefore, the risks of perforation and hemorrhages should be precisely assessed before treatment.

While although involved field radiotherapy (IFRT) was the most regular treatment option for NKTL patients with localized nasal involvement,^17^ three patients (10.0%) received radiotherapy in the GI‐involved cohort, and if imaging examinations and endoscopies indicate a high probability of the above, surgeries before chemotherapy treatment should be considered. A few previous retrospective studies showed patients with GI involvement rarely received radiotherapy.^22^, ^24^, ^25^ This might be due to the following: (1) the radiotherapy field was difficult to confirm because of multiple intestinal involvements; and (2) most patients were in advanced stages and local radiotherapy could not affect other lesions beyond those in the radiotherapy field.

The clinical efficacy of ENKTL was disappointing, especially in patients with extranasal involvement and advanced stages.^5^, ^17^, ^26^ In a retrospective study, patients with extranasal invasion who accepted HDT‐ASCT had a 2‐year OS of 60.1% and a 2‐year PFS of 54.5%, which were better than in previous studies,^27^ suggesting that HDT‐ASCT may improve the prognosis of patients with extranasal invasion. In our cohort, one patient in stage I achieved long‐term survival after receiving HDT‐ASCT as consolidation therapy. At the last follow‐up, both OS and PFS exceeded 36 months. It was suggested that the patients with GI involvement might benefit by HDT‐ASCT, which was consistent with the results above. Moreover, a proportion of relapsed patients could be effectively rescued with HDT‐ASCT.^15^ In summary, consolidation or a salvage role for HDT‐ASCT might exist for relapsed patients who achieve a second remission and extranasal patients who have achieved CR1.

Our study has limitations. Given the rarity of the disease and GI involvement being less common, this study was a retrospective study. In addition, the heterogeneity of individual characteristics and heterogeneous treatment modalities influenced the results, which highlights the need for further studies on this disease. Third, due to the small number of cases the multivariate survival analysis using COX regression model should be interpreted with caution.

## 5 CONCLUSIONS

Even through comprehensive treatment strategies, the prognosis of the disease remained poor, especially for patients of advanced stage. Asparaginase‐based chemotherapy may be beneficial to early‐stage patients with GI‐ENKTL. Of note, surgical intervention was also a feasible approach to deal with emergency complications. IPI and NRI seemed to be suitable for assessing the prognosis of patients with GI‐ENKTL. These results should be further validated by expanding the sample size in future studies and more effective treatment protocols such as HDT‐ASCT consolidation therapy were needed to explore. These findings helped choose appropriate treatment strategies in individual GI‐ENKTL cases.

## AUTHOR CONTRIBUTIONS

**Jiaxin Liu:** Data curation (equal); formal analysis (equal); writing – original draft (equal); writing – review and editing (equal). **Xin Liu:** Data curation (equal); formal analysis (equal); investigation (equal). **Yong Yang:** Conceptualization (equal); data curation (equal); formal analysis (equal); investigation (equal). **Weiping Liu:** Formal analysis (equal); investigation (equal); project administration (equal); resources (equal); supervision (equal); visualization (equal); writing – review and editing (equal). **Ying Wang:** Data curation (equal). **Xia He:** Data curation (equal). **Liling Zhang:** Data curation (equal). **Baolin Qu:** Data curation (equal). **Liting Qian:** Data curation (equal). **Xiaorong Hou:** Data curation (equal). **Xueying Qiao:** Data curation (equal). **Hua Wang:** Data curation (equal). **Gaofeng Li:** Data curation (equal). **Yuan Zhu:** Data curation (equal). **Jianzhong Cao:** Data curation (equal). **Junxin Wu:** Data curation (equal). **Tao Wu:** Data curation (equal). **Suyu Zhu:** Data curation (equal). **Mei Shi:** Data curation (equal). **Huilai Zhang:** Data curation (equal). **Hang Su:** Data curation (equal). **Yujing Zhang:** Data curation (equal). **Jun Zhu:** Funding acquisition (equal); project administration (equal); resources (equal); supervision (equal). **Shunan Qi:** Data curation (equal); formal analysis (equal); investigation (equal); project administration (equal); visualization (equal); writing – review and editing (equal). **Yexiong Li:** Funding acquisition (equal); project administration (equal); resources (equal). **Yuqin Song:** Conceptualization (equal); methodology (equal); project administration (equal); resources (equal); software (equal); validation (equal); visualization (equal).

## CONFLICT OF INTEREST STATEMENT

The authors declare no conflict of interest.

## ETHICS STATEMENT

The authors' institution provided ethical approval for the conduct of this study.

## INFORMED CONSENT STATEMENT

Informed consent was obtained from all individual patients for being included in the series.

## Data Availability

The datasets used and/or analyzed during the current study are available from the corresponding author on reasonable request.
